# Supplementary material for: Genetic comparisons of interleukin-17 reveal a framework for complex signaling evolution
Source: bioRxiv. 2026 Apr 14:2026.04.13.718218. Preprint. [Version 1] doi: 10.64898/2026.04.13.718218 (PMC13105070; doi:10.64898/2026.04.13.718218)
Supplement: Supplement 3 [file media-3.zip › Supplemental files for paper/ERC/IL17xIL17_comparisons/IL17REL_IL17RE.pdf]

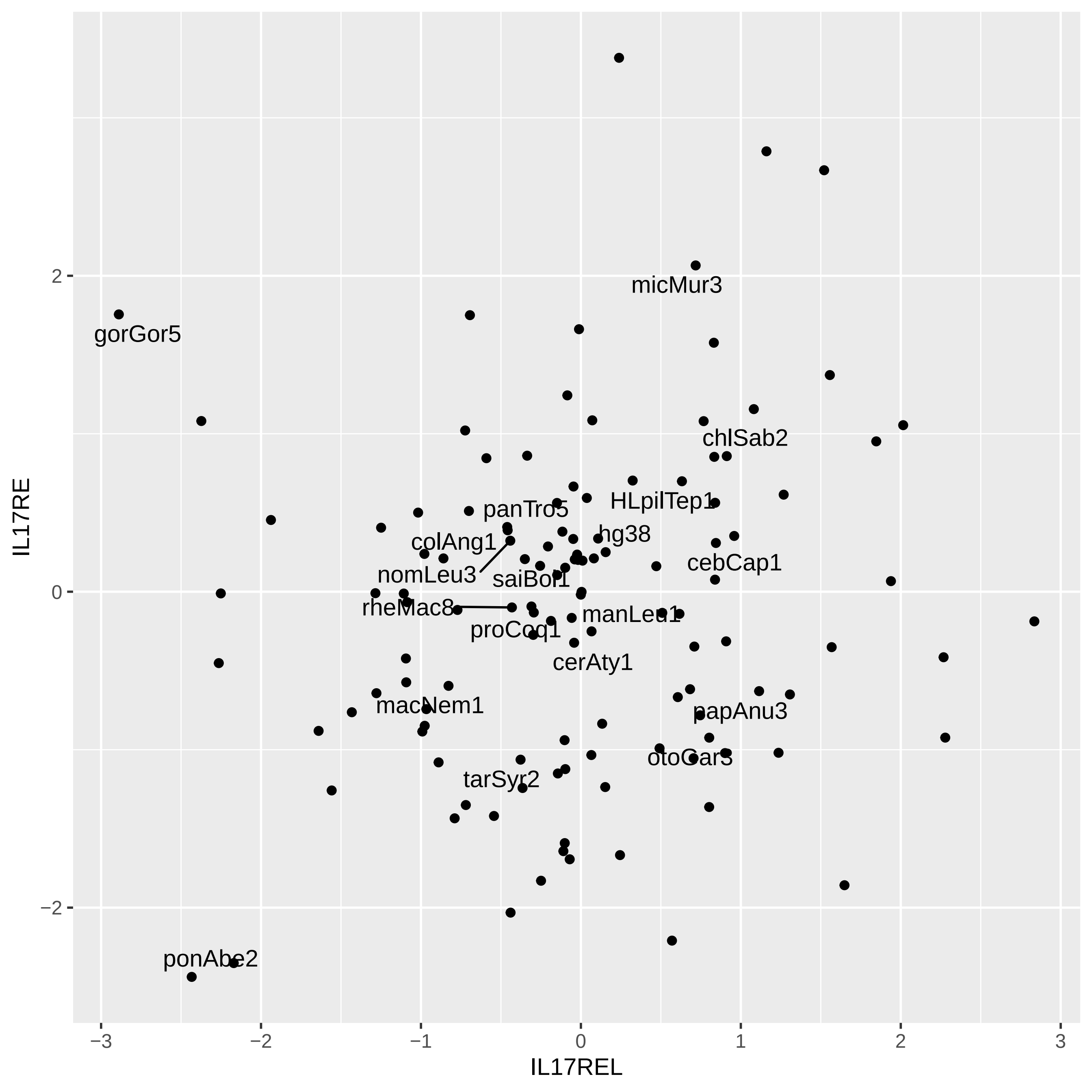

micMur3
gorGor5
chlSab2
HLpilTep1
panTro5
hg38
colAng1
cebCap1
nomLeu3
saiBol1
rheMac8
manLeu1
proCoq1
cerAty1
macNem1
papAnu3
otoGar3
tarSyr2
ponAbe2
IL17RE
IL17REL
2
0
−2
−3
−2
−1
0
1
2
3
